# The Hospital. Nursing Section

**Published:** 1903-11-14

**Authors:** 


### The Hospital.

### Nursing Section.

Contributions for this Section of "The Hospital" should be addressed to the Editor, "The Hospital" Nursing Section, 28 & 29 Southampton Street, Strand, London, W.C.

No. 894.—Vol. XXXV. *SATURDAY, NOVEMBER* 14, 1903.

### Notes on News from the Nursing World.

### IMPERIAL MILITARY NURSING SERVICE.

Miss Hannah W. Reid has been appointed one of the matrons of Queen Alexandra's Imperial Military Nursing Service. Miss F. M. Hodgins and Miss E. W. Hordley have been provisionally appointed to be sisters; and Miss A. L. Walker to be staff nurse.

### QUEEN'S NURSES FOR IRELAND.

The Countess of Dudley has just visited three of the districts in the West of Ireland, where she has placed nurses trained by Queen Victoria's Jubilee Institute. A remarkable evidence of the popularity of her Excellency's scheme was afforded in one of the districts. Not only was an address read to her expressing the gratitude of the inhabitants, but a bonfire was lighted opposite the nurse's cottage in honour of her visit. Wherever she went Lady Dudley was received with the utmost cordiality by the local clergy, rural councillors, and Poor-law Guardians. It cannot be doubted that the enthusiasm which the appointment of the nurses already established at Carna, Belladangen and Geesalla has excited will encourage the wife of the Lord-Lieutenant in her admirable efforts to supply one of the most urgent necessities of the poverty-stricken portions of the sister island.

### THE NURSES OF THE ROTUNDA HOSPITAL AND THEIR MASTER.

The retiring master of the Rotunda Hospital, Dublin, Dr. R. Dancer Purefoy, has been afforded the most convincing testimony of the feelings of esteem and gratitude which he has inspired among the nurses of the institution during his term of office. Last week, in the nurses' drawing-room at the hospital, a large company met in order to witness a presentation made to Dr. Purefoy by the nursing staff, of an address and a handsomely-bound album. Dr. Little, consulting physician to the hospital, who read the address and made the presentation, mentioned that among the many improvements effected by the master was a pathological laboratory built at his own expense. He went on to speak of the manner in which Dr. Purefoy had advanced the interests of the nursing staff, and mentioned that during his term of office it had been increased from 20 to 30. The address was signed by 213 nurses in all parts of the world, and Dr. Little observed that there could be no better evidence than this of the regard in which the master was held. It was subsequently stated by the chairman, who announced his acceptance on behalf of the Governors of a bust in bronze of Dr. Purefoy presented to the hospital by the nursing staff during his term of office, that 400 nurses had been trained under his auspices, each of whom, he believed, was indebted to him for the kindly counsel and advice he was always willing to give. Dr. Purefoy, in acknowledging the presentation, spoke in the warmest terms of his relations with the lady superintendent and the nursing staff, and said that the album would be one of the most highly-prized mementoes of his mastership.

### HOSPITAL-TRAINED NURSES AND POOR-LAW APPOINTMENTS.

The complaint of a workhouse infirmary-trained nurse that all the best Poor-law vacancies are filled by nurses trained in general hospitals, which is endorsed this week by another correspondent, who writes bitterly as well as briefly, does not surprise us. The fact which forms the subject of comment is indisputable. We have noticed that a great many of the best of the appointments in question have been filled in the manner described, and we sympathise with the numerous thoroughly well qualified infirmary-trained nurses who have the mortification of seeing themselves passed over. If we cannot concur in the suggestion that hospital-trained nurses should keep to hospitals, it is because we do not think it would be for the ultimate advantage of infirmary-trained nurses themselves that it should be acted upon. It must be remembered that until recent years the training at even the leading workhouse infirmaries was far inferior to that of the leading general hospitals, and the vast improvements which have been effected in many of the former are largely due to the introduction of hospital-trained nurses and hospital methods of nursing. We believe that as it becomes more and more clear that the great workhouse infirmaries are not inferior as training schools to the great general hospitals, the nurses who hold the certificates of the former will find that their applications for the best posts in the Poor-law service are not put aside in favour of those of "outsiders."

### MALE NURSES AT THE POPLAR SICK ASYLUM.

It is, we understand, quite true that six male nurses are to be engaged at the Poplar and Stepney Sick Asylum, if the Local Government Board consent. They are to receive salaries varying from 21s. to 26s. per week, with uniform and such meals as will fall in their time of duty. The managers have decided upon this step, owing to the statement of the medical staff that the work of attending "certain male patients" is altogether unsuited to women. Dr. Spurrell considers that male patients who are perfectly helpless, and whose weight is such that an average woman is not physically strong enough to do the necessary work, as well as those who persistently indulge in bad language, ought to be attended by male nurses.

### GROSS CARELESSNESS AT DUDLEY HOSPITAL.

A case of gross carelessness at the Guest Hospital, Dudley, has come under our notice, and as the authorities refuse to move in the matter, we think it necessary to call attention to it. Mrs. Giacinta M. M. Gallagher was trained at the hospital and passed her examination on June 2nd, 1903. The Secretary, on June 19th, in acquainting her with this fact, asked her to return a certificate which she had previously received as to her training, in order that it might be replaced by one which would include the passing of the examination. This request was complied with during the same week, but the new certificate was not forwarded for two months. Nurse Gallagher had then to write and point out that the date was wrong, the certificate testifying that she passed her examination on April 3, 1903, whereas her examination did not take place until June 2nd. She also drew attention to the circumstance that one signature was wanting, and that no mention was made of the one year's nursing she had done on the private staff of the hospital. After waiting a month without obtaining a reply, she wrote again, and received a communication from the Secretary to the effect that her letter had been read at a meeting of the Board, and that he was directed to say, in reply, that it was resolved "that it lie on the table." Meanwhile, the nurse had applied to the matron of a hospital which she wished to enter for midwifery training, but the matron, noticing the discrepancy between the date of the certificate and the nurse's statement as to when she passed her examination, wrote to the matron of the Guest Hospital, quoting the facts cited by the nurse, and asking for an explanation. The latter replied, "With regard to Nurse Gallagher, her statements are correct." There is, therefore, no possible justification for the action, or inaction, of the Board of the Guest Hospital, and it is clearly the duty of the subscribers to that institution to demand an inquiry into the circumstances. Their strange hesitation to rectify a mistake, which might have prevented one of their nurses from gaining employment elsewhere, cannot fail, if persisted in, to damage the reputation of the hospital as a training school.

### THE TRAINING OF NUNS AS NURSES.

There is one cause for congratulation with respect to the scandal at Granard. It has prompted the medical officer of Carlow Dispensary, while taking up the cudgels on behalf of the nurses who were employed at the infirmary, to suggest that it might be an advantage for one sister in each religious community to go through the ordinary course of hospital work and receive her nursing certificate. This, at any rate, would be a step in the right direction, and in acting upon it the communities in Ireland would only be following the admirable example of the managers of the Hospital of St. John of Jerusalem in London. The objections to nursing by nuns, whether in Ireland or elsewhere, so far as all reasonable people are concerned, would disappear entirely, if they usually possessed, in addition to high personal qualifications for nursing, the training which some have enjoyed.

### "NAZARENE NURSES."

Yet another order of nurses—this time in the United States. A Roman Catholic association of women in Brooklyn has organised a class for what they term "Nazarene Nurses," to train mission workers to care for the sick poor of "Greater New York." The course is only to comprise ten weeks of study, and it is stated that no attempt will be made in that period to teach more "than the rudiments of looking after the sick in order that the settlement workers and missionary assistants may be enabled to aid the physicians in their ministrations to destitute patients." We are afraid that the title of "Nazarene Nurses" will give rise to mistaken impressions as to the nursing knowledge of the settlement workers and missionary assistants.

### UNFOUNDED CHARGES AGAINST AN INFIRMARY NURSE.

Public bodies are sometimes rather prone to listen to tittle-tattle about their officers. A case in point was brought before the Richmond Board of Guardians the other day by the Rev. E. C. Cleal, chairman of the Visiting Committee, who stated that they had considered a communication in which charges were brought against one of the nurses. After careful investigation the committee came to the conclusion that there was no foundation for the charges, which arose out of a letter sent to Miss Foster Newton from a lady who visited the workhouse. The committee having decided to investigate the matter, the person making the accusations was invited to attend, in order to substantiate her statements. The charges were that the nurse had tampered with the food, made use of language unbecoming to a nurse, and used considerable violence. The person making the charges, however, refused to attend, and the committee decided to deal with the allegations stated in the letter in the presence of the doctor, the superintendent nurse, and her assistant. It appears that they sat for two hours, and after hearing all the evidence, unanimously determined that there was no ground whatever for bringing the charges. The board unanimously decided to write to Miss Newton, acquainting her with the committee's decision, Mr. Dimbleby remarking that she could in no way be blamed for communicating the charges, which had been made to her by an inmate. But the least the correspondent who forwarded the complaint to Miss Newton can do is to apologise to the nurse for formulating accusations for which there does not appear to be a shadow of foundation.

### GRATUITY TO THE MATRON OF A WORKHOUSE INFIRMARY.

A gratuity of £20 has been awarded by the St. George's-in-the-East Board of Guardians to Miss Wesley, matron of the infirmary. The reason for the gratuity was explained at the last meeting of the guardians. Miss Wesley has been in the service of the guardians for 14 years, and attained her maximum salary in 1891. The guardians have since wished to increase it, and charge it to the common poor fund, but the Local Government Board decided that it could only be augmented by way of gratuity. There is no question of the burden of the ratepayers being rendered more heavy, as the subscription of £20 which the guardians used to pay for the purpose of obtaining trained nurses from an association not now in existence has been saved since the matron of the infirmary undertook the duty. But we agree with the guardians that it would be much more satisfactory if, instead of voting a gratuity, the salary of the matron were permanently increased by £20 and made a charge upon the common poor fund, and we hope that the further attempt to obtain the sanction of the Local Government Board which is contemplated will be successful.

### ZENANA BIBLE AND MEDICAL MISSION.

An Indian exhibition and sale was held on behalf of this Society at the Portman Rooms on Thursday and Friday last week. There was a fair attendance on the first day, when the exhibition was opened by the Duchess of Somerset, who was accompanied by Lady Muriel Paget. Lord Kinnaird presided at the opening ceremony, and the Hon. Gertrude Kinnaird explained that the object of the gathering was to draw attention to the great needs of India's women. She said that some years ago a hospital for women was built at Nasik by some wealthy Hindoos, but in consequence of the difficulty of obtaining reliable doctors the work had not been satisfactory, so that these Hindoos had, through their missionary, asked the society to take over the hospital, even if they conducted it on Christian lines. The Society now appealed for a fully-qualified lady doctor for this hospital. Another was also required for a hospital for Indian women at Ajodhya, near Lucknow, which had hitherto been under the care of a native woman. The Duchess of Somerset, in declaring the exhibition open, said that it was a pleasure and a privilege to her to be present, and she hoped that the work would receive the support it so richly deserved. Among the special features of the exhibition was a small model of the Lady Kinnaird Memorial Hospital, one of the three maintained by the Society in India. This hospital has accommodation for 50 in-patients. During 1902 the lady doctors provided by the Medical Mission attended 23,219 cases, and at the dispensaries 72,921.

### THE DISTRICT NURSING QUESTION AT CHELTENHAM.

In consequence of an incorrect report of the proceedings at a meeting of the Cheltenham Board of Guardians in a local paper, which our contemporary now explains was due to "confusion of voices," we were led to suppose that the Cheltenham District Nursing Association had not treated the Guardians with the consideration which the latter, as generous contributors to the organisation, merited. This, however, we are glad to hear, is not the case. It appears that from the outset the Guardians left it entirely to the committee of the District Nursing Association to plant the nurses where they thought fit. The Association in placing one at Bentham were influenced by the immense difficulty experienced in finding suitable rooms, and would not have chosen that village if they could have fixed upon a better centre. This autumn they were able to secure her rooms at Leckhampton, which is three, not six, miles from Bentham, and as here most of her work lies, the removal, which was arranged at headquarters and not by the nurse, was regarded as an improvement. The only fault to be found with the Association seems therefore to be that they omitted, by an oversight, to advise the Guardians of the nurse's removal, and for this they have apologised. The Guardians have since referred the question to the House and Dispensary Committee, and we trust that they will arrive at a determination which will prevent any break in the work the Association have carried on happily in Cheltenham for years.

### THE DIETARY AT NEWTON ABBOT.

There have been so many complaints about the nurses' rations at Newton Abbot Workhouse Infirmary that the details of the dietary may be read with interest. Seven pounds of bread are allowed each nurse during the week; six pounds of meat, beef, mutton, pork, all parts and served in various ways; three and a half pints of milk to drink and for puddings, $\frac{3}{4}$ lb. of butter, 1 lb. of bacon, $\frac{1}{2}$ lb. of cheese, four ounces of tea, three ounces of coffee, three eggs, $\frac{3}{4}$ lb. of loaf sugar, $\frac{3}{4}$ lb. of moist sugar, a cake not less than one pound, $\frac{1}{2}$ lb. of biscuits, and five pounds of potatoes. Vegetables are supplied from the garden as required, a pot of jam once a month, and a pudding or tart every day. Fish has been applied for and granted.

### CONCERTS FOR NURSING ASSOCIATIONS.

An interesting movement has been inaugurated in Somersetshire during the winter, and up to the end of May a series of concerts will be given in different villages, the proceeds of which will be divided between the Bridgwater District Nursing Association and Village Nurse Funds. The first two of these entertainments have just been held at Quantock Lodge, the residence of Mr. Stanley, the member for the division, and his wife, and in the parish schoolroom at Over Stowey. In each instance there was a large attendance, and the substantial sum of £21 was received at the doors. As the concerts will in most cases be given at private houses, the whole of the takings will practically be devoted to the admirable purpose in view.

### OUR CHISTMAS DISTRIBUTION.

We have received the following contributions to our Christmas distribution of clothing, in addition to the parcels already acknowledged:—R.N.P.F. 101, Miss Alice M. Hitchcock, The Beeches, Avenue Road, Lynn, Norfolk, and Nurse Besant, 4 York Street Chambers, W.

### SHORT ITEMS.

Many of our readers will doubtless be glad to know that "Hints on How to Start a District Nursing Association," which has lately appeared in our columns, will shortly be published in book form by the Scientific Press, Limited, 28 and 29 Southampton Street, at the small sum of one shilling.—A sale of the patients' work at the British Home and Hospital for Incurables, Streatham, will take place in the hall of the institution on Wednesday next. It will be opened by Lord Grenfell at 11 a.m.

### Some Practical Points in the Nursing of Diseases of the Skin.

By Norman Meachen, M.D., B.S., M.R.C.P.Lond., M.R.C.P.Edin., Physician to the Skin Department, Tottenham Hospital, N.

(*Concluded from page 67.*)

*Varicose eczema* of the lower extremities is a very common complaint, and is frequently the precursor of ulceration. The influence of the force of gravity, combined with the congestion associated with the varicose veins in those who have to stand for many hours a day, renders the condition a difficult one to treat unless the limb can be placed in a horizontal position. When the acute stage has passed, one of the most favourite applications is zinc glycerine jelly, usually known as Unna's paste. When you are called upon to apply this form of dressing, do not look upon the process merely as a daubing of the skin with a brush in the same way as a man tars a fence. If you do, the result will be—failure. Rather regard it as a scientific means of assisting Nature in her efforts to heal. First carefully cleanse the limb all round its circumference, wherever it is intended that the paste should go, with a 2 per cent. solution of lysol, wiping away all traces of old ointment, loose scales, dirt, and débris with swabs of cotton-wool. While this is being done, the gelatin-paste, which is obtained in cubes, should be melted in a small earthenware vessel (*e.g.*, a jam-pot) in a saucepan of water over the fire. Just before you are ready to paint the limb, dab it lightly with a pledget of wool moistened with ether, care being taken to see that no lamp or candle is near at the time. The paste should be of the consistency of thick cream and just pleasantly hot. It must be applied with a large, clean paste-brush, the strokes being made slowly and deliberately in a direction transverse to the long axis of the limb. When the whole circumference has been thickly and evenly painted over, wait for five minutes and then dab the coat of paste all over with a piece of dry cotton wool, some of the fibres of which will stick to the paste, thus giving greater body to it, besides affording the additional advantage of allowing it to dry sooner. This dressing is cleanly, comfortable, and it can be kept in place for several days. When it is desired to inspect the skin, the coat of paste can be rolled off in one piece, if it has been well applied, after the manner of a glove.

*Ulcer of the leg* is a natural pathological result of long-continued congestion. The mistake most often made in dressing these painful sores is to envelope the whole circumference of the leg around the ulcer with a rag saturated with ointment. To obtain the best results, the size and contour of the ulcer should be accurately gauged with the eye, and a piece of lint cut the exact shape. This must be spread on the smooth side with the ointment prescribed and fitted into the hole. On no account must the edges of the ulcer be covered in with the same dressing which is applied to its base. In the same manner, if the ulcer be a large one, with several "islands" of skin appearing as whitish dots upon the raw base, these must not be covered in by the ointment, but small holes should be made in the lint corresponding to their position.

Both in ulcer and eczema of the leg careful bandaging is a helpful adjunct to recovery. The bandage should be preferably one of the "open" elastic varieties such as those made of "crêpe." The dark rubber ones must be carefully eschewed. It should be applied in the classical way, "from within outwards," commencing at the inner border of the instep, with a figure of 8 around the ankle, and then by firm even "reversings" ascending as high as the knees.

*Psoriasis*, when very abundantly distributed upon the body, is often admitted into hospital wards for various treatment by baths, tar, etc. The bath should be warm enough to allow of the patient remaining in it for ten minutes without getting chilled, from 90° to 98° Fahr. being a convenient temperature. The medicament having been added, the patient should soak the scaly patches well, and, on emerging from the bath, she must be dried quickly and gently, the ointment being then freely rubbed into the places. When the disease affects the scalp, a shampoo with soft soap to which a little spirits of wine has been added will materially assist in removing scales and preparing the way for the application of ointment.

New patches or alterations in the shape or appearance of old ones, or the occurrence of other lesions, such as blisters or vesicles, when noticed by the nurse whilst dressing or washing patients should, of course, be reported to the doctor at his next visit.

*Ringworm of the Scalp* so often begins as a tiny scaly patch which is only too often passed over as simple "scurfiness." When this terrible disease has taken a firm foothold it is very difficult to eradicate. Never neglect, therefore, to report at once the appearance of a small scaly place in a child's head, as, if vigorous treatment is begun early, an epidemic of ringworm, which is a serious thing in a children's ward, may by your promptitude be prevented. You will not be expected to diagnose ringworm from other affections of the hair, but once you have seen a good many cases and learnt to recognise the peculiar stumpy hairs—as if they had been nibbled by a mouse, or resembling stubble in a cornfield—you are pretty sure to be right in saying that a scaly patch on the scalp is one of ringworm if these appearances be present. You have, in all probability, had experience of shaving the scalp for wounds, so it will not be an unusual thing if you should be required to shave a wide area round the affected patch. The ointment prescribed must be rubbed well in twice daily with a small stiff brush, a new tooth-brush will do excellently. Any fresh places must be attacked in the same manner. Should you have the misfortune to contract the disease upon your hands or face as a result of attending to a child suffering with ringworm, you have the consolation of knowing that tinea of the body is much more speedily cured than when it affects the scalp, which is very rare in adults. Directly a small, reddish, itching spot appears, it should be at once painted with a little tincture of iodine, if upon the hands or arms, or if the face be attacked, a little of the white precipitate ointment should be rubbed over it.

The condition of the skin has such an important practical bearing upon the bodily health in general, and it is liable to be so greatly affected in many diseases that you can hardly be too often upon the look-out for the numerous signs that appear on the skin and which afford sometimes a valuable index as to the state of the patient and the progress of his disease. With many of these cutaneous symptoms you are familiar, such as the herpes of the lip in pneumonia, the red patches upon the limbs in certain forms of Bright's disease, the rashes occurring after enemata, and so on.

I trust that by these few remarks the interest among nurses in this important, if less attractive, branch of their art may be aroused and quickened.

### The Prevention of Bed-sores.

### EXAMINATION QUESTIONS FOR NURSES IN THE COLONIES AND ABROAD GENERALLY.

THE question was as follows set in April last:—"State what precautions you would take for the prevention of bed-sores."

### Criticism of Answers.

"Pietersburg" and "South Africa" send papers of nearly equal merit, but "Pietersburg's" is much more clearly expressed. I should, however, like to see greater pains taken with the literary part of this work, such as the turn of the sentences, the condensation of much information into little space, and the careful writing and spelling of the answer itself. I would ask all our candidates abroad to read carefully my remarks on the English competitors' work a fortnight ago as want of space prevents my repeating them. The nurses abroad, like those at home, seem hardly to realise adequately that impeded circulation is the chief factor in the formation of a bed-sore. Certainly strict cleanliness should be maintained, but washing ten times a day would be useless without relieving pressure and without prolonged and steady friction whereby circulation is restored. "South Africa" wisely objects to the use of powder and advocates the use of spirit and oil mixed together.

### First Prize.

To prevent bed-sores a nurse should first pay particular attention to the cleanliness of the skin, and see that there is no undue pressure on any part of the body caused by uneveness of sheets or mattress. There are not many feather-beds at this day, but one meets with them occasionally in old-fashioned houses.

If a nurse should find a patient on one, her first duty would be to persuade him to have it changed for a good firm mattress which would allow of sheets and draw-sheets being drawn lightly across. The under-blanket, sheet, and draw-sheet should be tucked well under the mattress on both sides, so that they will not continually be wrinkling up under the patient. The back and hips should be well washed with soap and water (using plenty of soap) at least three times in the 24 hours. The skin should be thoroughly dried, and then rubbed with brandy or methylated spirit, and dusted over with finely-powdered starch, or starch, zinc, and boric acid in equal parts. Lanoline well rubbed in keeps the skin nourished and the rubbing is also beneficial as helping to keep up the circulation.

Care should be taken to keep the patient quite dry, and the back should be looked at each time the bed-pan is used and, if necessary, the sheet drawn.

In cases of incontinence, it is well to keep the back and hips thickly smeared with zinc ointment, this allows the urine to run off. The sheets should be changed as often as possible, always using clean ones. Never use seamed or patched sheets for underneath a patient, it is better to put them on the top if they must be used.

Not only back and hips must be carefully watched for any sign of redness, but also shoulder-blades, elbows and heels.

Little ring-pads made of cotton-wool may be used for protecting the heels, but should be avoided for the back as they would probably do more harm than good, but if the patient is very thin and the sacrum is prominent, a circular air-cushion may be used for a few hours a day to relieve pressure.

Where the nature of the case allows of moving, the patient should not lie in one posture for more than two hours at a time, but be moved from side to side, a pillow against the back will prevent rolling over again.

In cases which must be kept continuously on the back, the nurse should get an assistant to raise the patient whilst she examines by means of a hand-mirror, and washes and powders quickly and draws the sheet.

In such cases, also, the night-dress should always be slit up the back as far as the yoke to ensure everything being perfectly smooth. PIETERSBURG.

### Second Prize.

To prevent bed-sores thorough cleanliness must be strictly adhered to regarding both patient and bed-linen. The patient's bed should be properly and comfortably made, linen changed regularly, kept dry and clean, especially the draw-sheet, which must never be allowed to crease under patient's back, neither must crumbs be permitted to collect. In South Africa, during the intense heat, it is essential to sponge patients at least once daily for comfort and cleanliness' sake; the back should be carefully washed twice daily, or more often if needed, with good plain soap and warm water, then dried thoroughly whilst the soap still remains on. Afterwards back, shoulders, and hips should be rubbed with methylated spirit and olive oil (two parts of spirit and one of oil) and gently massaged; dusting powder I never use in this climate. From long practical experience I have proved washing with good plain soap, followed by gentle massage, to be the best preventive amongst all remedies against bed-sores, particularly for patients who may be confined to bed for several months. Precautions must also be taken with continued serious cases to move them from one position to another (with the medical officer's permission) in order that too much pressure may not be confined to any one part of body or limbs. In serious prolonged cases I daily take the same precautions with feet and heels as I use with shoulders, back, and hips, as these are the most tender parts which receive greatest pressure. Another important precaution for the prevention of bed-sores which requires constant consideration is feeding the patient with stimulating and nourishing food, otherwise he gradually grows weaker, blood becomes poor and circulation gets bad. In such cases bed-sores are almost certain to arise if the above necessary precautions are not strictly adhered to in time. Sunshine and fresh air are most essential to patients, they each help to cheer and brighten and keep them in good spirits and so they enjoy good nourishing food, and often prevent patients feeling depressed, from which so many complications frequently occur. SOUTH AFRICA.

### Honourable Mention.

This is gained by "ZZZ," "A Probationer," and "Viking," who all send good papers. "A Probationer" contributes some interesting information with regard to the Cingalese, who, she says, suffer but little from bed-sores, even in prolonged illness, in consequence of the toughening of their skin from constant exposure and the use of various oils.

### Defective Papers.

Some candidates still cling to the dangerously deceptive collodion. If it is agreed that friction is all important, how is it to be carried out with a glassy surface of this material? "Bulawayo" makes a serious mistake in advocating rubbing gently with "the tips of the fingers!" Never use the tips, but always use the whole hand slightly curved like a cup, and rub steadily with a circular movement for several minutes.

### Question for April.

State what curative treatment you should pursue in cases of slight and also of serious bed-sores.

All answers must reach this office not later than March 20th, 1904. The rules are as below, except that *six months are allowed* in which to send in papers instead of the fifteen days granted to nurses in the United Kingdom.

N.B.—This question is not for nurses in England.

THE EXAMINER.

### Rules.

The competition is open to all. Answers must not exceed 500 words, and must be written on one side of the paper only. The pseudonym, as well as the proper name and address, must be written on the *same* paper, and *not on a separate sheet.* Papers may be sent in for fifteen days only from the day of the publication of the question. All illustrations strictly prohibited. Failure to comply with these rules will disqualify the candidate for competition. Prizes will be awarded for the two best answers. Papers to be sent to "The Editor," with "Examination" written on the left-hand corner of the envelope.

N.B.—The decision of the examiner is final, and no correspondence on the subject can be entertained.

In addition to two prizes honourable mention cards will be awarded to those who have sent in exceptionally good papers.

### Workhouse Nursing.

### THE HOSPITALS ASSOCIATION AND THE REPORT OF THE DEPARTMENTAL COMMITTEE.

(*Concluded from page 82.*)

Paper by Miss Gibson (*continued*).

Here I must touch on the vexed question of the relative positions of the matron and the superintendent nurse. I put the master out of my mind, because he must be the administrative head of his whole establishment, and in all matters of discipline be supreme. But the superintendent nurse's obedience is due only to the medical officer, and so long as she conforms to the disciplinary rules of the house her wards and her sick should be her own domain, in which only the medical officer and the Guardians have any authority. But in a small place it is not possible that two people can hold the positions of matron and superintendent nurse respectively, as they exist at present, unless they are both very exceptionally full of tact.

I know that I shall be told that a "wise matron does not interfere," and this seems to me a most feeble argument. If the matron is in charge of the sick wards, and she does not interfere, she neglects her duty; and surely the Local Government Board do not desire to call those officials wise who do not do what they are expected to do. It is no more right towards the matron than towards the superintendent nurse. If the superintendent nurse is the responsible person, she ought not to have any possibility of interference; if she is not, then the workhouse matron, it seems to me, under existing circumstances, is bound to interfere. The position is anomalous, and ought not to continue. To make a superintendent nurse, as she is at present, responsible to the Guardians, the medical officer, the master, and the matron. is simply encouraging friction. Is it likely that, unless she is for some reason inferior, she will be willing to be under the authority of one who is not her superior in knowledge, and who, besides, is not a nurse? Would anyone, who was not absolutely lacking in ability or ambition or enthusiasm for her profession, remain in this position? I am sure not, and therefore, and not for any other reason, I support the proposal of the committee that the superintendent nurse be made an independent official, subject to the medical officer as to her work, and in administrative matters to the master. I feel sure that, if this is not done, the best type of nurse—the type whom we, I am sure, all desire to see caring for our poor, imbuing their probationers with enthusiasm for their work and love for the very poorest and most ill, the type who love human beings more than cases, and who are willing to spend and be spent in the service of the poor—will shake the dust of Poor-law nursing off their feet, not because they wish to give it up, but because its conditions are unbearable.

The Threatened Decadence of Poor-Law Nurses.

It has to be remembered that in these days there are many outlets for those nurses who wish to nurse the poor; the Jubilee Institute swallows up a large number of the very best type and has an almost insatiable appetite for more, and district nursing provides all that is dear to the heart of the true Poor-law nurse, and leaves her, besides, a free woman, answerable only to her medical officer and her committee. Private nursing also tempts many, though these are largely of another kind, and everything which tends to reduce the responsibility and the enthusiasm of the Poor-law nurse tends to lessen the supply and to lower its quality. How can we prevent this threatened decadence of Poor-law nurses? How can we improve on existing conditions? I think in two ways: first, by doing our very best to impress on the Local Government Board the necessity and the wisdom of forming a special department to deal with this question of nursing; and second, by using every argument and every endeavour to secure the appointment of women inspectors, who are nursing experts, to visit and inspect and report on the nursing of the small unions.

Women Inspectors.

On the first of these suggestions—the formation of a special department—I cannot but believe that some day it will be seen how reasonable is this idea of women inspectors.

If we could only become less parochial—only understand that nursing the sick poor is a question for the whole country, and not only for each individual parish—that we are working for a common cause! When we have planted the idea of the common good, perhaps the central department will not be far off. To be of service, this department should be made up of the medical and some of the lay inspectors or officials of the Local Government Board, two or three women who are experts in nursing, and two or three chairmen of infirmary committees. Their function should be, shortly, to choose candidates for training and to draft these for a fixed period to the large infirmaries. Dr. Downes deprecates the increase of probationers in large infirmaries, and instead wishes to bring them into minor ones where, he says, they will "expel stagnation and quicken the life of a workhouse sick ward." But as he also says that "in the minor training school they are to learn the first steps in their calling under trained and competent women," it is difficult to understand why there should have been "stagnation," unless there was not enough work to keep the "trained and competent women" alive; and if so, how can there be enough for two, and what knowledge and experience can the probationer gain? It all resolves itself into the old difficulty. You cannot make bricks without straw, and you cannot train nurses without material. The nursing experts on my proposed general department would deal with this and all other illusions, and would, I believe, expel them.

I have left but little time for the question of women inspectors, but fortunately there is little difference of opinion on this subject. The regular visitation of all country unions by a woman who is an experienced nurse, and who is also well versed in administration and the needs of the poor and sick, would be of inestimable value, not only to the inmates themselves, but also to nurses isolated in remote places. Such inspectors would actually see the nursing done, would give help and advice, see that necessaries were supplied, and help nurses in a hundred ways to bear monotony and to give their best interest to their work.

The Necessity for Speaking Out.

This is only a meagre sketch of what I want to say. I do not speak unadvisedly. I have given twenty years of my life to the study of poor-law nursing. If the nursing of your workhouses is to be kept up, if, in fact, there is not to be one class of nurse for the rich and an inferior class for the poor, it is necessary that we should speak out now. The pauper suffers as keenly as the prince, and needs, and should have, the same care. Help us to give the best we have in nursing to those who cannot demand it and who need it so sorely.

This help will only be given, and poor-law nursing will only remain as it should be, if the nurses who are in its service are trained thoroughly and on the best nursing lines. There is a certain discipline of work and conduct, a certain professional manner and method of work, which every probationer in a hospital or large infirmary goes through, which goes very far to teach her her true attitude to her patients, to her work, and her fellow-workers. This training of mind is most necessary and desirable, and the probationer who has been grounded well in a good school starts with a serviceable stock-in-trade which it will be difficult for her to gain later. It seems to me imperative that all nurses should begin in a school where hospital discipline and hospital methods are insisted upon. This would, of course, be impossible except in an organised hospital or infirmary. And I will conclude by urging upon you the necessity of teaching all nurses on nursing and not on workhouse lines, remembering that we must all be made nurses first, and Poor-law officers afterwards.

Miss Baker, a Guardian of the Holborn Union, said that already the report had borne bad fruit. The Board of which she was a member had already started one of these minor schools. They had one superintendent nurse, four charge nurses, 12 assistant nurses, one head night nurse, and one head ward nurse. That staff was supposed to be able to train probationers and turn them out qualified nurses at the end of one year. They could not possibly give the time to the requisite bedside training.

### WORKHOUSE NURSING—*Continued.*

Mr. W. Parkinson, master of St. Olave's Workhouse, said that he agreed with Dr. Savill in objecting to the so-called "qualified nurse." He did not suppose that any master or matron advocated the scheme, and he had some authority for saying that it would never be put in force.

Dr. J. T. Macnamara, medical officer of Ladywell Workhouse, thought the Local Government Board was moving in the right direction in trying to raise the tone of Poor-law nursing and trying to assimilate the training to that of the general hospitals. But the stumbling-block was the attempt to establish the minor schools—a most retrograde step. In his institution, with 700 inmates, they had one nurse superintendent, and not one of the other nurses was fully trained, while under them were a lot of attendants not trained at all. When the matron and superintendent retired there was no nurse in charge at all. That was the kind of thing the Association was trying to alter. The superintendent nurse must be put under the direction of the district medical officer and must be given absolute control of the sick wards, whether the master and matron liked it or no.

Mrs. Despard, a Guardian of Lambeth, dealing with the question of friction between matrons and superintendent nurses, said that it was certainly not the fault of the latter. It was the inevitable result of putting a person untrained in a certain capacity in authority over one very elaborately trained in that particular respect, and that was a difficulty the Local Government Board never foresaw.

Dr. A. Scott, of Braintree, said that they were making progress: ten years ago at his place they had one trained nurse and the patients were really nursed by paupers. Now they had probationers—as respectable a class of young women as they could get, and they gave them the best training they could.

Mrs. T. Paston Brown stated that in the infirmary with which she was connected, with from 250 to 270 inmates, they had one superintendent, four assistants, and the rest paupers, and, as might be expected, anything more terrible than the state of the wards she had never seen. To-day they had a staff of 30 nurses, and were in a building distinct from the workhouse. That meant a great deal, and she felt that the day on which the door was shut between the workhouse and the infirmary was the proudest day of her life.

Mrs. Bedford Fenwick, though the hour was getting late, asked to be allowed to say a word in admiration of the papers to which they had listened and to emphasise the need for a standard of nursing. The great training schools ought to co-operate to state what a trained nurse was. As things were, the period might be three months, one year, or three years. The whole country was flooded with inefficient and unqualified nurses. They wanted a bill introduced into Parliament for an organised nursing council which should settle the curriculum, the standard of qualification, and the registration of nurses.

Dr. Savill then moved the following resolution :—

"That this meeting disagrees with the proposal of the Departmental Committee on Nursing to create minor training schools in small workhouses, and the recommendation that a probationer who has undergone at least one year's training in such schools should be considered and called a "qualified nurse." This meeting believes that the adoption of these recommendations would have a most detrimental effect upon the position and character of nursing as a profession, and an equally injurious influence on the Poor-law service and on the public interests. It would ask the President of the Local Government Board to receive a deputation.

The resolution was put to the meeting and carried *nem. con.*

Colonel Montefiore proposed and Dr. Macnamara seconded a vote of thanks to the Treasurer of St. Thomas's Hospital for the use of the hall, which Mr. Wainwright acknowledged.

### Central Midwives Board.

At a meeting of the Central Midwives Board—Dr. F. H. Champneys in the chair—held at the Board-room, 6 Suffolk Street, S.W., on October 29th, and by adjournment also on November 5th, the following business was transacted :—

### An Impostor.

A letter was read informing the Board that a person representing himself as deputed by the County Councils to receive the names and fees of midwives desiring to be certificated by the Central Midwives Board had visited a nursing institution and had endeavoured to collect the fees. The Board desire it to be known that the secretary alone is authorised to receive the fees, and as they are in possession of a description of the impostor, they trust that the publicity of the press may lead to his early detection.

### The Colonial Nursing Association.

A letter was read from the Colonial Nursing Association directing the attention of the Board to the case of the midwives now working abroad, sent out by the Association. As most of them were in distant parts of the world, it would not be practically possible for them to forward their certificates to the secretary for verification. Under the special circumstances of these cases the Board resolved to adopt the procedure prescribed by the rules in the case of lost certificates, and to accept the voucher of the secretary of the certifying institution that such a certificate had in fact been granted to the applicant, together with a certificate of identity, as a substitute for the production of the original certificate.

### Approved Qualifications.

The Secretary was instructed to add the Rural Midwives Association, 47 Victoria Street, S.W., to the list of bodies to whom applicants for training in midwifery should be referred. After consideration of applications received in each case, the Board resolved to receive the certificates of the following bodies as approved qualifications under Section 2 of the Midwives Act, 1902 :—Queen Charlotte's Lying-in Hospital; Manchester Southern and Maternity Hospital; Liverpool Ladies' Charity and Lying-in Hospital; British Lying-in Hospital; Glasgow Maternity Hospital; and St. Mary's Hospital, Manchester. The consideration of applications from various bodies for recognition as institutions approved by the Board, under Section C. of the rules, and from certain qualified medical practitioners for approval as teachers under the same section, was deferred until the board should be in a position. to formulate their scheme of examination.

### Names on the Roll.

Draft rules of procedure on proposed removal of a midwife's name from the Roll, or cancelling of her certificate, were considered, amended, and ordered to be forwarded to the Privy Council for approval. After consideration of applications for certificates, the names of 126 women were passed under Section 2 of the Act, and ordered for entry on the Roll. Of this total 74 claimed as holding the certificate of the Obstetrical Society of London, three that of the Rotunda Hospital, Dublin, and 49 were admitted as having been in *bonâ-fide* practice for one year prior to July 31st, 1902. A design of certificate on parchment was approved and ordered to be printed. A form of certificate of identity, as prepared by the secretary, was approved and ordered to be placed on sale with the other official forms of the Board.

### The Register of Cases.

After consideration of tenders for printing and binding the Register of Cases [Rule E. 19 (a)], that of Messrs. Spottiswoode and Co., Limited, 54 Gracechurch Street, E.C., and 5 New Street Square, E.C., was accepted. The Register can be obtained from Messrs. Spottiswoode, as above, price 1s., postage extra. The secretary reported that as the Privy Council had raised no objection to the Board issuing their "Suggestions to County and County Borough Councils" on their own responsibility, he had sent out copies to all those Councils—about 50 in number—who had applied for them.

### New Books on Nursing.

A Case Book for Private Nursing. (London: The Scientific Press, Limited, 28 and 29 Southampton Street, Strand. 1903. 46 pages. 3d. net).

Every well-trained private nurse takes a pride in her "report" and will spend much time in compiling a neat and legible record for the doctor. Ordinary case books published have often been found too involved and too small to be of much practical help. Therefore, most nurses arrange their own. Many a private nurse can recall much time, especially at night, spent in trying to rule lines that would smear and go crooked, or in endeavouring to "print" headings, and of general disgust and dismay—particularly in the early morning light—over the miserable-looking chronicle when there was no time to rewrite it. Now, however, there need be no more trouble, and this admirably-arranged book should supply a long-felt want. It is the size of an exercise book—7½ inches by 9½ inches—and is neatly bound in a grey cover. The paper is good, it is well ruled, and the headings are clearly printed. The book is arranged for day and night nurses' reports, and the columns are divided in a sensible way which is taken in by the eye at a glance. Private hospitals, nursing homes, schools and orphanages should all find this case book of value, and the publishers make special quotations for a quantity. There will in future certainly be no excuse for any private nurse who shows a doctor an untidy or jumbled report, when, for the price of an ordinary exercise book, she can provide herself with so neat and valuable a register. Nurses are judged a good deal by their "reports"; and a private nurse, armed with a carefully-kept temperature chart and this latest case book, will help the doctor very considerably.

### Presentations.

Brownlow Hill Infirmary, Liverpool.—Miss M. E. Hime, who has been appointed matron of Mildmay Cottage Hospital, London, has been presented by the nurses and probationers of Brownlow Hill Infirmary, Liverpool, which she entered eleven years ago, finally becoming assistant superintendent, with a silver tea-service; by the matron with a Gladstone bag; and by the night superintendent with a silver photograph frame.

Park Hospital, Hither Green.—Miss Elizabeth Palmer, who for the last three-and-a-half years has been assistant matron at the Park Hospital, Hither Green, on leaving to take up her duties as matron to St. Anne's Home, Herne Bay, was presented by the nursing staff with a handsome silver toilet requisites, and by the domestic staff with a leather travelling-bag and silver-mounted Wedgwood hot-water jug as a token of their esteem and good wishes for her future success.

Swansea Hospital.—Miss Letitia Bown was presented on November 7th, on her resigning her post of sister of the Llewellyn Ward, Swansea Hospital, by the nurses and patients, with a handsome silver-backed brush, a tortoise-shell silver-mounted comb, a silver-mounted scent bottle, and by her fellow-sisters with a silver-backed hat brush, as a token of their esteem and respect.

### Official Announcements.

THE NURSES' CO-OPERATION.

We print the following interesting and instructive announcement just as we received it:—

The nurses on the staff of the Nurses' Co-operation present their compliments to the Editor of The Hospital, and trust that he will spare space in his paper for the accompanying notice.

November 11th, 1903. 8 New Cavendish Street, W.

A pleasant little ceremony took place on the 6th inst. at the Howard de Walden Nurses' Home, when Miss Clutton was the guest of the Nurses' Co-operation. As a Member of Committee Miss Clutton has been connected with the Co-operation since the very early days of its existence, and for many years she also held office as Hon. Treasurer, devoting much time and thought to the administration of the financial department.

When Miss Clutton recently resigned the treasurership, the nursing staff determined to show their appreciation of her labours, and as a result of this decision they invited her to an "At Home" on Friday afternoon. Tea was served in the restaurant, which is a distinctive feature of the Home, and in which, be it noted, the nurses are able to obtain comfortable meals at any hour. More than a hundred nurses were present on Friday, and the majority of the staff, prevented by duty from attending, regretted their enforced absence from the pleasant gathering.

Some members of the committee were also guests on the occasion, and a few friends kindly gave an excellent programme of music later on, when the piano, given to the Home by Mrs. Large (*née* Miss Philippa Hicks), the first lady superintendent of the co-operation, was much appreciated.

The presentatian to Miss Clutton was made in the clubroom, which was gay with flowers, and Nurse Janet Christie, on behalf of the nurses, said: "Miss Clutton, we are very pleased to welcome you here this afternoon, and we wish, first of all, to express the regret we all felt on learning that we were to lose your very valuable services on the committee. We desire also to thank you most cordially for the good and capable work you have done for us; for your untiring energy and interest in us during many years you have been our hon. treasurer. Although your health demanded rest, you stuck to us until we were well started in this comfortable home, and no one was more pleased than yourself to know that everything was paid for, even to the return of the loan made us by Lady Howard de Walden, and which we were not actually bound to pay off for some twenty years to come. However, we share your pride that we are free from any obligation, and that our society is in a most flourishing condition both financially and otherwise. Indeed, we nurses think, that in spite of reports which have been occasionally spread to the contrary, we may well boast that there is no other Nurses' Institution more flourishing, nor one in which there exists a better spirit of loyalty—amongst ourselves, and to our committee; moreover we do not feel any fear that we shall lose the position to which we have attained.

"We are very glad to have Miss Baker for our Home-Sister—she is one of ourselves—and we feel confident that she will do everything in her power to make us comfortable, and that this Home will work just as smoothly under her management as the offices of the Co-operation do under our lady superintendent, Miss Roberts."

Miss Christie concluded by asking Miss Clutton's acceptance of a very beautiful silver entrée-dish, "As a little token of the nurses' gratitude." Miss Clutton received the gift with many expressions of pleasure, and assured the nurses that her interest in their welfare had by no means ceased. Although no longer taking an active part in the administration of the business of the Society, she remained a member of the Co-operation."

### Appointments.

Cobham District Nursing Association.—Miss Annie Mattinson has been appointed nurse. She was trained at the Manchester Royal Infirmary, where she was afterwards staff nurse. She has since been charge nurse at the fever hospital, Barnsley, and the Carmarthenshire Infirmary, and district nurse at Ipswich.

Cumberland Infirmary, Carlisle.—Miss C. Richardson has been appointed sister. She was trained at the Bristol Royal Infirmary, and has since been sister of the gynæcological and ophthalmic wards, with the charge of two theatres attached.

Hackney Union Infirmary, Homerton.—Miss Mary Emma Covington and Miss Helena Jane Mooney have been appointed charge nurses. Miss Covington was trained at Lambeth Workhouse Infirmary, and has since been attached to a nursing institute at Harrow-on-the-Hill and at Woodford Green. Miss Mooney was trained at Swansea General and Eye Hospital, and has since been charge nurse at Long Reach Hospital, Dartford, and nurse-matron at Hucknall Torkard Hospital, Notts.

Hitchin Workhouse Infirmary.—Miss Alice Flick has been appointed assistant nurse. She was trained at the Invalid Asylum, High Street, Stoke Newington, by the Meath Workhouse Nursing Association.

Huddersfield Union Deanhouse Workhouse.—Miss Catherine Mohan has been appointed charge nurse. She was trained at Sculcoates Union Infirmary, where she subsequently became assistant nurse.

Infectious Diseases Hospital, Portsmouth.—Miss F. Petchey has been appointed matron. She was trained at the Fountain Fever Hospital, Tooting, S.W., and the Royal Hants County Hospital, Winchester. She has since been home sister and housekeeper at the Royal Hants County Hospital, Winchester.

Keighley and District Hospital. — Miss Eleanor Constance Laurence has been appointed matron. She was trained at the Sick Children's Hospital, Great Ormond Street, Bloomsbury, and Guy's Hospital, London. She has since been ward sister and night superintendent and assistant matron at Guy's Hospital, and lady superintendent of Princess Christian's Hospital in South Africa during the Boer war.

Lancaster District Nursing Association.—Miss A. Gurd has been appointed nurse in charge. She was trained at Edinburgh Royal Infirmary and Belfast Maternity Hospital, and had district training at the Portsmouth Home. For the last four years and a half she has worked as a Queen's Nurse. She holds the L.O.S. certificate.

Mildmay Cottage Hospital, London.—Miss M. E. Hime has been appointed matron. She was trained at the Workhouse Infirmary, Brownlow Hill, Liverpool, where she has since been charge nurse, night superintendent, and assistant superintendent.

Raikes Moor Hospital, Barrow-in-Furness.—Miss Audrey Thurston has been appointed charge nurse. She was trained at St. Saviour's Infirmary, East Dulwich, and under the Metropolitan Asylums Board at their fever hospitals. She has been night nurse at Fulham Infirmary, charge nurse at Willesden Isolation Hospital, temporary nurse at the Evan Fraser Small-pox Hospital, Hull, charge nurse and assistant matron at Blawirth Hill Hospital, Yoker, and Matron of the Isolation Hospital, Draycott, near Derby.

St. Leonard's Infirmary, Shoreditch.—Miss Edith Cavell has been appointed assistant matron. She was trained at the London Hospital, Whitechapel, E., and has since been night superintendent at St. Pancras Infirmary.

Sheffield Royal Hospital.—Miss Ethel G. Hall has been appointed sister. She was trained at the General Hospital, Birmingham, and has since been nurse at Netley House Surgical Home, Cavendish Square, London, at the Nursing Home Mentone.

### Everybody's Opinion.

### ASEPTIC FLOORING FOR OPERATING THEATRE.

"S. L." writes: Would any of your kind readers with knowledge of the usage and durability of aseptic flooring for the operating theatre give me the benefit of their experience in the following?—Our theatre floor is of Portland cement, which is very unsatisfactory in being porous, rough, and gritty. Mosaic has been suggested, but I believe that also is more or less porous at the joints, has a tendency to crack, and therefore becomes septic. This is a small hospital and our funds being low, cost has naturally to be carefully considered.

### THE ANGLO-AMERICAN NURSING HOME AT ROME.

"The Patient" writes: I see in your paper for October 17th that "The Nurse" writes criticising the expressions used by an "English Nurse" in a former letter which was inserted in your columns. As I happen to be both the patient and the nurse referred to in that letter, and as I nursed my nurse and friend through her attack of typhoid, I feel as if I knew something about the circumstances, and should like to have a little "say" in the discussion now going on. It is not necessary for me to comment on the unkindness and neglect shown by the matron and committee of the Anglo-American Nursing Home towards a nurse who fell ill while in their service, as an "English Nurse's" letter speaks graphically for itself. All I would point out is the lack of common courtesy and consideration shown towards both, patients and nurses alike. I should like "A Nurse" to explain the term "a German," which she uses in her criticism of an "English Nurse's" letter. I was always under the impression that if a nurse, whatever her nationality may be, holds the nursing certificate of one of the largest London hospitals she is at perfect liberty to sign herself an "English Nurse" without asking her less fortunate comrade's permission to do so."

### THE TRAINING OF MIDWIVES IN RURAL DISTRICTS.

"Mrs. Rickman" writes from 12 Airlie Gardens, Campden Hill, W.:—May I be allowed to say a few words on the rather "astonishing" comment on my so-called "astonishing speech" in your issue last week. I am personally (and so, of course, is the Rural Midwives Association) most anxious that every woman who undertakes the work of a midwife shall be fully trained, as now, thank God, under the Midwives Act, she soon must be. At the same time I think the work should be left to the class of women who, when fully-trained, are able so successfully to do it, as was so conclusively shown in the paper read by Miss Wilson. Is it right or just that women of higher class and education should compete with these women for earnings which will at the best only amount to £75 a year? This sum Miss Gregory gave as the possible, not probable, earnings of a midwife working amongst the poor. £50 to £70 a year is not the market value of a trained, educated woman, and if she works for such a sum she must be considered as giving a portion of her services in charity. The only time allotted to me as the representative of the Rural Midwives Association in which to state my views was two minutes, and this may account for the mistake that has arisen.

### Echoes from the Outside World.

### The King's Birthday.

In celebration of the anniversary of the King's birthday the bells of the Curfew Tower of Windsor Castle and of the parish church of St. John were rung on Monday morning. A Royal Salute of 41 guns was fired by X battery of the Royal Horse Artillery in St. James's Park. Flags were flying from the Admiralty and various other public buildidgs at Westminster. At Sandringham his Majesty, as usual, gave a dinner to the workmen on his estate, about 600 in number. The King, the Prince of Wales, his Royal Highness's children, and the house party visited the tent while the dinner was in progress. The anniversary was observed in various parts of the country, and at night the principal thoroughfares in the West End of London were brilliantly illuminated.

### Royal Betrothal.

It was officially intimated on Tuesday that Princess Alice of Albany is betrothed to Prince Alexander of Teck. Princess Alice is the daughter of the Duchess of Albany, and is 20 years of age. Prince Alexander is the youngest son of the late Duke of Teck, the brother of the Princess of Wales, and he is in his thirtieth year. He served in the Matabele and the South African wars, gaining the Distinguished Service Order. He is a Knight Commander of the Royal Victorian Order.

### Memorials to Queen Victoria.

Excellent progress has been made with the National Memorial to Queen Victoria in front of Buckingham Palace. The thoroughfare leading into Birdcage Walk is practically completed, and little more remains to be done in widening and shaping the semi-circular space in front of the King's residence, and in the centre of which the great statue of Queen Victoria will stand. Last week a fresh stage was entered upon, when the removal of several rows of trees in the Mall was commenced in preparation for the construction of the new avenue, 65 feet broad, which is to run down the centre. It is intended that the new thoroughfare shall run straight up to the statue of the Queen, so that there shall be an uninterrupted view from beyond the Duke of York's steps.

A beautiful statue of the Saviour, about 17 feet high, has been erected on the right of the steps of the approach to the Royal Mausoleum, Frogmore, at the expense of the Queen, in memory of Queen Victoria, with the inscription "The best of mothers-in-law." The statue was sent from Denmark.

### The Birthday Honours.

The list of Birthday Honours conferred by the King was published on Monday. There are no new peers, but baronetcies are conferred on Colonel John E. Bingham, twice master cutler of Sheffield, and Mr. Lees Knowles, M.P., honorary secretary to the Guinness Trust for Housing the Poor. The new knights, in addition to Mr. John George Craggs, honorary secretary of King Edward's Hospital Fund since its foundation, and Mr. Alan Manley, surgeon-apothecary to the King and Prince of Wales, include Mr. R. K. Douglas, a famous Chinese Scholar, Mr. Charles Holroyd, keeper of the Tate Gallery, Mr. August Manns, musical director of the Crystal Palace since 1853 and founder of the Saturday Concerts, and Professor Clement Le Neve Foster, an expert on mines. In addition to other distinctions which have been conferred, his Majesty has been pleased to grant the Imperial Service Medal to 176 members of the Civil Services of the Empire on their retirement after 25 years, or more, of meritorious service.

### Lord Mayor's Day.

In accordance with custom, Lord Mayor's Day was celebrated in London by a great civic procession which, however, differed from most of its predecessors in the complete absence of emblematic cars and kindred symbolic displays, for which were substituted half a score of detachments from Metropolitan Volunteer corps. The crowds of spectators in the streets were as great as usual. In the evening a banquet took place at the Guildhall. The Prime Minister, who was the principal speaker, devoted himself almost entirely to external affairs. He declared that if it could not be affirmed that the diplomatic sky was cloudless, he could at least say that there was nothing in the present state of the world's affairs which need cause any overpowering anxiety; and in a subsequent passage, Mr. Balfour dwelt on the beneficial results of the King's visits to Portugal, Italy, and France, and of the return visits of the heads of these States.

### Death of Lord Rowton.

The death of Lord Rowton, in his sixty-sixth year, from pnuemonia following on influenza, took place on Monday morning at his house in Berkeley Square. Lord Rowton, who as Mr. Montagu Corry was private secretary to the late Lord Beaconsfield from 1866 to 1880, was in recent years, better known as the founder of the Rowton Houses, which are practically cheap hotels for poor single men of all classes in London. In fact, the last twenty-three years of his life were almost entirely devoted to philanthropic work. He was chairman of the Guinness Trust as well as of the Rowton Houses Company, and he was also on the committee of King Edward's Hospital Fund. To his hands were entrusted the papers of Lord Beaconsfield, and for some time it was anticipated that he would be the biographer of his former chief. Lord Rowton was unmarried and the peerage becomes extinct.

### Administration of the War Office.

It is announced that the Prime Minister, with the King's approval, and after consultation with the Secretary of State for War, has appointed a Committee to advise as to the creation of a board for the administrative business of the War Office, and as to the consequential changes thereby involved. The names of the committee are Viscount Esher (chairman), Admiral Sir John Fisher, and Colonel Sir George Sydenham Clarke. Lord Esher has had considerable experience of Government departmental work in the capacity of Permanent Secretary of the Office of Works, and was early in his career, as Mr. Reginald Brett, private secretary to the Duke of Devonshire, then Lord Hartington. Admiral Sir John Fisher has served as one of the Lords of the Admiralty, and was recently appointed to the Portsmouth command. Sir George Sydenham Clarke is at present Governor of Victoria, and will not resign, but only temporarily cease to occupy, his post at the Antipodes.

### A Kite-boat Voyage.

The inventor of the war kite, Mr. S. F. Cody, made an experimental voyage on Saturday which was sufficiently successful for him to claim to have established the practical value of his invention for purposes of navigation under favourable conditions of wind when other means of transit are not available. The kite is connected to the boat, which is 14 feet in length, partially covered with canvas, and unsinkable and self-righting. The former was maintained at an altitude of about a quarter of a mile during the trip across the Channel. Mr. Cody left Calais at eight o'clock on Friday evening. The night was light and the sea was moderate, but the air was very cold. The wind was so "fluky" that Mr. Cody had frequently to use his sea-anchors to moderate the pace of his boat. He once narrowly escaped being run down by a steamer bound up Channel. He sailed into Dover at half-past eight on Saturday, the kite having then been hauled in and attached to the masts in order that it might act as a sail.

### For Reading to the Sick.

WATCHING UNTO PRAYER.

Who knows? God knows: and what He knows
Is well and best.
The darkness hideth not from Him, but glows
Clear as morning or the evening rose
Of East or West.

Wherefore man's strength is to sit still:
Not wasting care
To antedate to-morrow's good or ill;
Yet watching meekly, watching with goodwill,
Watching to prayer.

*C. Rossetti.*

It may be that the clouds in your horizon are, even now, causing you to tremble. But do not fear; God will come with them, and the hand that has led you will still lead you on. Do not let the thought of to-morrow's cross overshadow the Lord's present love to you. For what does anxiety about to-morrow do? It will not empty to-morrow of its sorrow, but it does empty to-day of its strength. These trials are sent to mould you more and more into the likeness of Christ. Nothing you have is half so much as what you are. Do not look at to-morrow's cross, but cast yourself unreservedly upon Him and His love. Not one crested wave shall break over you that does not bring with it its own blessed recompense. "He maketh the clouds His chariot, and walketh upon the wings of the wind."

*Rev. F. Whitfield.*

If suffering endues the Christian with the "spirit of glory" even here below, the consequence must be a change in the whole of his being. He will feel, as successive troubles and trials sweep over him, that not only are they bearing him on to the haven where he would be, but that he is gaining through them a something which he never could have gained under the calm sunny skies of a happy life. "Your life," says St. Paul, "is hid with Christ in God." It is the life of the spirit which is thus hidden, the life which is shared with Christ now, which is a part of Himself. "The God of all patience" knows what we are suffering, and He will wait. He only asks that the bruised and wearied heart should lie passive for a while beneath His rod. If we are His children, and wish, however feebly, to honour Him, He will carry us in His bosom, and He will lead us. The same God who bore patiently with Elijah's breaking heart will bear with us; let us but lie still, the message will surely come to us at last as it came to the prophet in the wilderness, and strength will be given.

*Mrs. Gordon Campbell.*

There is a secret in the ways of God,
With His own children, which none others know,
That sweetens all He does; and if such peace,
While under His afflicting hand, we find,
What will it be to see Him as He is,
And past the reach of all that now disturbs
The tranquil soul's repose? To contemplate,
In retrospect unclouded, all the means
By which His wisdom has prepared His saints
For the vast weight of glory which remains.

*Swaine.*

### Notes and Queries.

**FOR REGULATIONS SEE PAGE 21.**

*Urine Testing.*

(57) Can you recommend a little book on "Urine Testing" suitable for a nurse?—*Nurse C.*

A new work on this subject entitled "The Examination of the Urine," by Dr. J. K. Watson, has just been published by the Scientific Press, Southampton Street, Strand, price 1s. 1d. post free.

*Abroad.*

(58) I should like to know whether an English certificated nurse can obtain nursing in Hamburg or Antwerp, and if it would be an advantage to have the L.O S. certificate.—*Nurse Marie.*

Only through private introductions. The L.O.S. is always a useful certificate to hold, especially abroad.

Please tell me the names of one or two private nursing institutions abroad through which I might obtain work? I was trained at University College Hospital.—*Nurse B.*

Consult "The Nursing Profession: How and Where to Train," published by the Scientific Press.

*Male Nurse.*

(59) Can you tell me where I can get free training as a male nurse?—*A. C. T.*

The National Hospital for the Paralysed and Epileptic, Queen's Square, Bloomsbury, W.C., is the only hospital in Great Britain where male nurses are trained. Some asylums for the insane afford their attendants opportunities to acquire a certain amount of training in the sick wards.

*Home.*

(60) Can you tell me of a home or institution where a young lady mentally enfeebled could be received by paying a few shillings a week. She is not insane, but she has met with accidents which have weakened her brain power.—*A. B.*

Apply to the Secretary, the National Association for Promoting the Welfare of the Feeble-minded, 53 Victoria Street, S.W.

*Thyroid Tabloids.*

(61) I should be glad to know if there is any risk attached to taking thyroid tabloids in myxœdema; if there are any precautions to be taken, and how soon a result may be looked for.—*M. S.*

Consult the medical man in charge of the case.

*Infectious Cases.*

(62) Is it safe to visit cases of diphtheria and measles whilst nursing ordinary district cases?—*District Nurse.*

The care of infectious and non-infectious cases must not be combined.

*Society of Chartered Nurses.*

(63) Will you kindly tell me how I can become a member of the Chartered and Registered Association of Nurses?—*M. H. R.*

Are you not confusing two societies? Apply to the Secretary of the Registered Nurses' Society, 269 Regent Street, W., for particulars of the one, and to the Secretary of the Society of Chartered Nurses, 24 Prince's Street, Cavendish Square, W., for particulars of the other.

*Training.*

(64) I am desirous of getting a few months' training in nursing the sick poor, and I should be much obliged if you will tell me if it would be possible for me to get such training under a district nurse in some country village or town, as I am not strong enough to go through a course of training in an institution?—*Puzzled.*

It would be quite possible to arrange a course of training under a district nurse privately, through the matron of a nursing home. Your letter is dated Bournemouth; possibly the Lady Superintendent, the Victoria and Bournemouth Nurses' Institute, Inverness House, could help you to obtain your wish.

**Important Nursing Textbooks.**

"The Nursing Profession: How and where to Train." 2s. net 2s. 4d. post free.

"A Handbook for Nurses." By Dr. J. K. Watson. 5s. net; 5s. 4d. post free.

"Practical Guide to Surgical Bandaging and Dressings." By Wm. Johnson Smith, F.R.C.S. 2s. post free.

"The Nurses' Dictionary of Medical Terms and Nursing Treatment." By Honnor Morten. 2s. post free.

"The Human Body: its Personal Hygiene and Practical Physiology." By B. P. Colton. 5s. post free.

"Art of Feeding the Invalid." (Popular Edition). 1s. 6d. post free.

"On Preparation for Operation in Private Houses. By Stanmore Bishop, F.R.C.S. 6d. post free.

